# Supplementary material for: Comparative analysis of methods to reduce activation signature gene expression in PBMCs
Source: Sci Rep. 2023 Dec 28;13:23086. doi: 10.1038/s41598-023-49611-2 (PMC10754832; doi:10.1038/s41598-023-49611-2)
Supplement: Supplementary file 1 — Supplementary Legends. [file 41598_2023_49611_MOESM1_ESM.docx]

Title: **Comparative Analysis of Methods to Reduce Activation Signature Gene Expression in PBMCs**

Authors: Lovatiana Andriamboavonjy^1,2,+^, Adam MacDonald^3,4+^, Laura K. Hamilton^1^, Marjorie Labrecque^1^, Marie-Noёlle Boivin^5^, Jason Karamchandani^5,6^, Jo Anne Stratton^3,4*^, Martine Tetreault^1,2,7*^

**Supplemental Information**

**Supplementary Figure S1: Adding TTi cocktail lyses red blood cells requiring addition of RBC lysis buffer for cell concentration determination**

**A** Schematic of the experimental plan for PBMC isolation with and without (+/-) RBC lysis buffer at RT (n=2/condition).

**B** Volcano plots of transcriptomic differences between PBMCs isolated at RT +/- RBC lysis buffer all without TTis showing 28 DEGs, with 25 down-regulated and 3 up-regulated.

**C** Volcano plots of transcriptomic differences between PBMCs isolated at RT +/- RBC lysis buffer all with addition of TTis showing 274 DEGs, with 149 down-regulated and 125 up-regulated.

**D** Schematic of experimental plan for PBMC isolation (+/-) RBC lysis buffer and cold.

**E** Volcano plots of transcriptomic differences between PBMCs isolated cold +/- RBC lysis buffer all without TTis showing 45 DEGs, with 35 down-regulated and 10 up-regulated.

**F** Volcano plots of transcriptomic differences between PBMCs isolated cold +/- RBC lysis buffer all with addition of TTis showing 21 DEGs, with 15 down-regulated and 6 up-regulated.

Error bars represent mean +/- SEM. Significance was set at p-value < 0.01 and an absolute log2FoldChange value of 0.58 (1.5 fold). Refer to TableS1 for raw transcriptomic data and TableS2 for complete DEG lists.

**Supplementary Figure S2: KEGG and GO analysis of down-regulated DEGs by cold processing or TTis show convergence on AP-1 complex.**

**A** KEGG pathway analysis of the 92 DEGs specifically down-regulated by cold processing predominantly identified ribosomal-related pathways.

**B** Gene ontology (GO) cellular compartment (CC) analysis of the 92 DEGs specifically down-regulated by cold processing revealed significant enrichment ribosomal-related processes.

**C** KEGG pathway analysis of the 216 DEGs down-regulated by addition of TTis predominantly identified cell contact and cell cycle-related pathways.

**D** Gene ontology (GO) cellular compartment (CC) analysis of DEGs down-regulated by addition of TTis revealed significant enrichment platelet and cytoskeletal-related processes.

Error bars represent mean +/- SEM. Significance was set at p-value < 0.01 and an absolute log2FoldChange value of 0.58 (1.5 fold). Refer to TableS1 for raw transcriptomic data and TableS2 for complete DEG lists.

**Supplementary Figure S3: KEGG pathway and GO enrichment analysis of DEGs between t0, t2, t24 hours post thaw.**

**A** KEGG pathway analysis of the 2256 DEGs down-regulated between t2pt and t0pt.

**B** Gene ontology (GO) cellular compartment (CC) analysis of the 2256 DEGs down-regulated between t2pt and t0pt.

**C** KEGG pathway analysis of the 2722 DEGs up-regulated between t2pt and t0pt.

**D** Gene ontology (GO) cellular compartment (CC) analysis of the 2722 DEGs up-regulated between t2pt and t0pt.

**E** KEGG pathway analysis of the 1041 DEGs up-regulated between t24pt and t0pt.

**F** Gene ontology (GO) cellular compartment (CC) analysis of the 1041 DEGs up-regulated between t24pt and t0pt.

**G** KEGG pathway analysis of the 1228 DEGs down-regulated between t24pt and t0pt.

Error bars represent mean +/- SEM. Significance was set at p-value < 0.01 and an absolute log2FoldChange value of 0.58 (1.5 fold). Refer to TableS1 for raw transcriptomic data and TableS2 for complete DEG lists.
