# Supplementary figures and images for: Comparative analysis of methods to reduce activation signature gene expression in PBMCs
Source: Sci Rep. 2023 Dec 28;13:23086. doi: 10.1038/s41598-023-49611-2 (PMC10754832; doi:10.1038/s41598-023-49611-2)

Figure S1

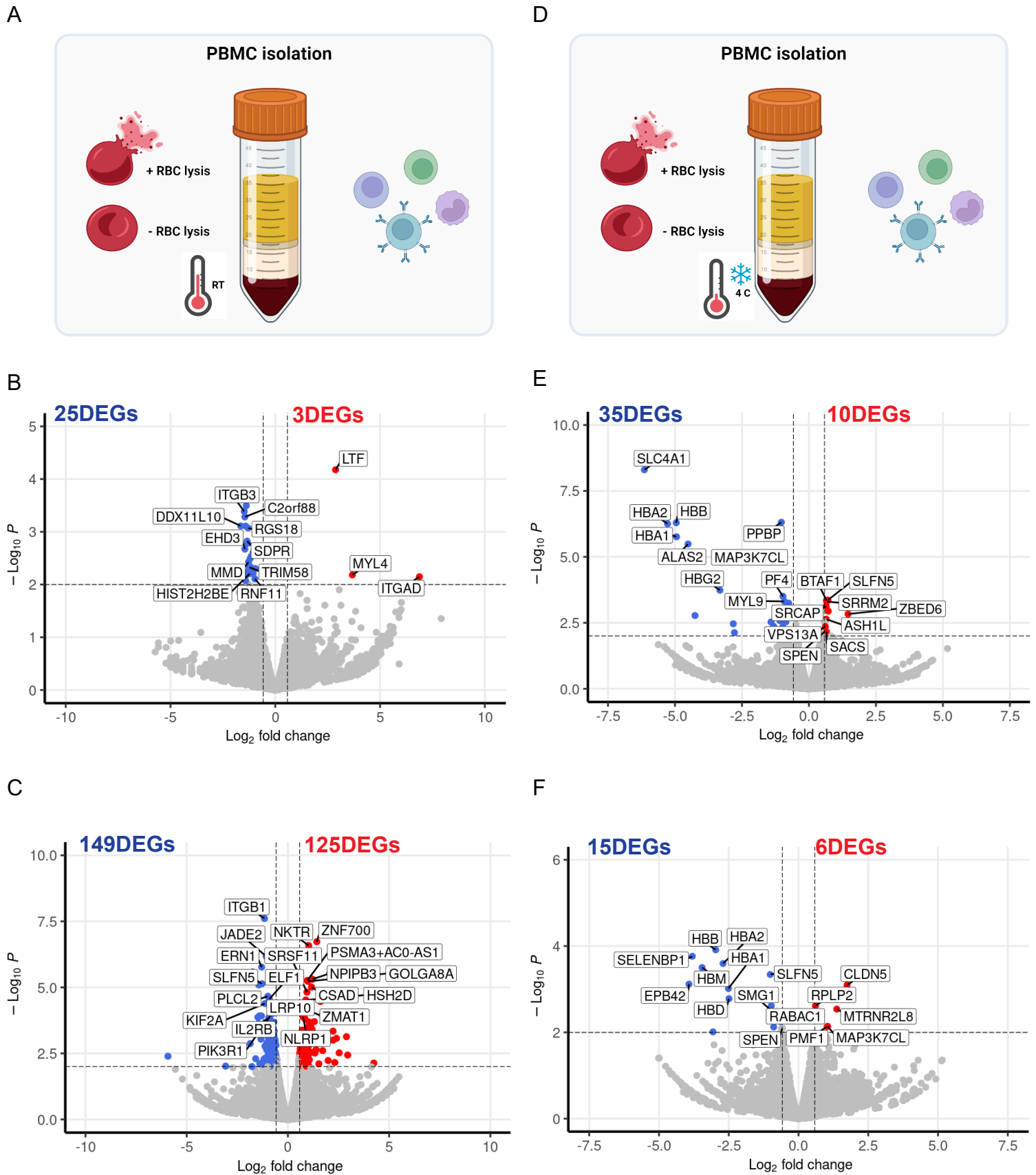

Supplement: Supplementary file 2 — Supplementary Figure S1. [file 41598_2023_49611_MOESM2_ESM.pdf]

Figure S2

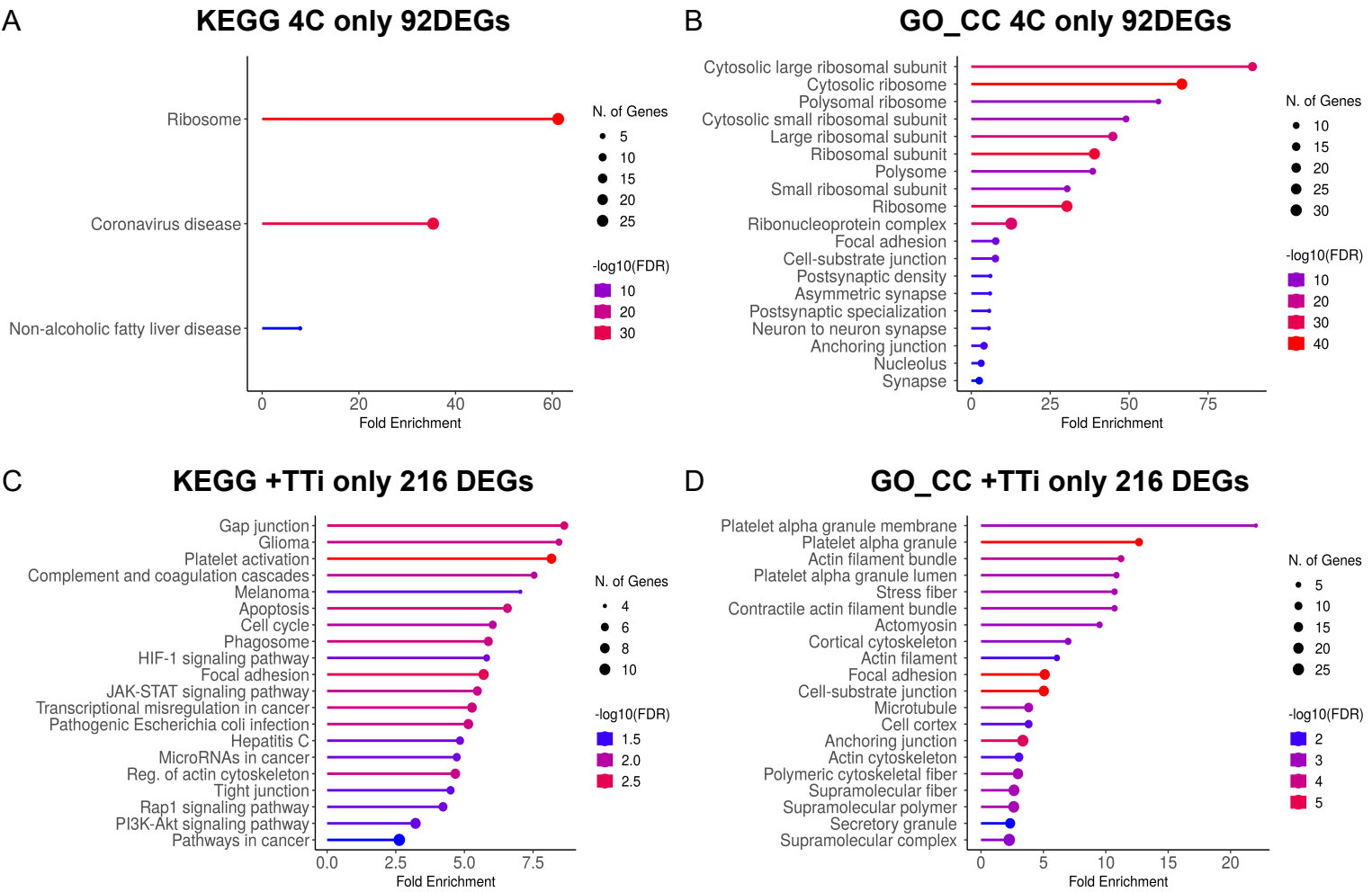

Supplement: Supplementary file 3 — Supplementary Figure S2. [file 41598_2023_49611_MOESM3_ESM.pdf]

Figure S3

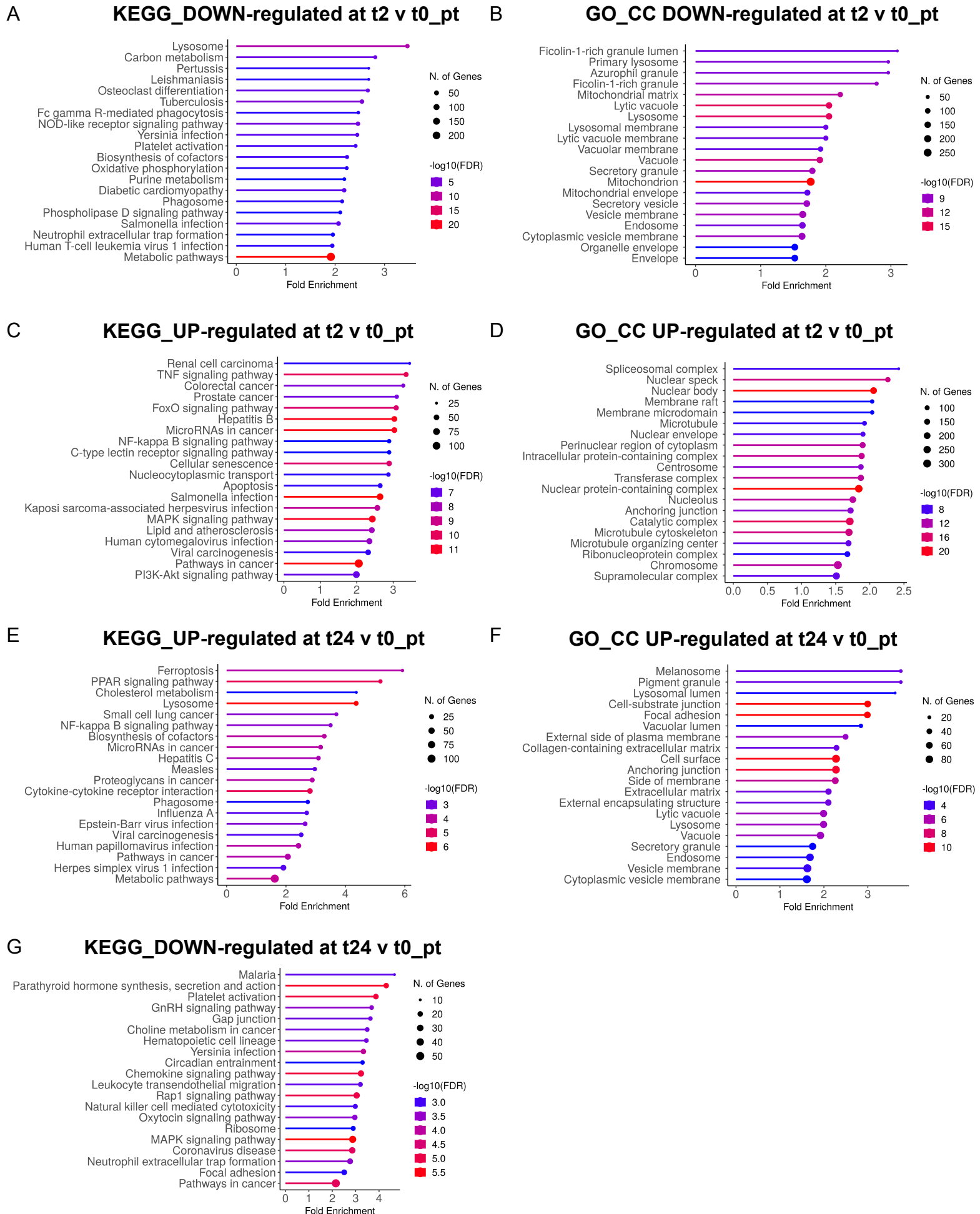

Supplement: Supplementary file 4 — Supplementary Figure S3. [file 41598_2023_49611_MOESM4_ESM.pdf]
